# Prognostic value of platelet to lymphocyte ratio in patients with colorectal cancer undergoing chemotherapy: a systematic review and meta-analysis

**DOI:** 10.3389/fimmu.2025.1642181

**Published:** 2025-11-03

**Authors:** Yanfang Li, Juan Zhou, Hui Luo, Shaohua Li, Yanru Shi, Di Chen, Xuehui Hu

**Affiliations:** ^1^ Department of Gastroenterology, Xijing Hospital, The Fourth Military Medical University, Xi’an, Shaanxi, China; ^2^ Department of Cardiovascular Medicine, The First Affiliated Hospital of Yangtze University, Hubei, Jingzhou, China; ^3^ Department of Psychiatry, Xijing Hospital, The Fourth Military Medical University, Xi’an, Shaanxi, China; ^4^ Department of Culture and Art Studies, Basic Medical Science Academy, The Fourth Military Medical University, Xi’an, Shaanxi, China; ^5^ Department of Nursing, Xijing Hospital, The Fourth Military Medical University, Xi’an, Shaanxi, China

**Keywords:** platelet to lymphocyte ratio, colorectal cancer, chemotherapy, prognostic value of survival, meta-analysis

## Abstract

**Background:**

Emerging evidence suggests a correlation between the platelet-to-lymphocyte ratio (PLR) and the prognosis in patients with colorectal cancer (CRC) undergoing chemotherapy. Nevertheless, the existing findings remain contentious.

**Methods:**

An extensive literature review was carried out using PubMed, Embase, Web of Science, and the Cochrane Library up to February 20, 2025, to identify relevant studies on the prognostic role of PLR in clinical outcomes. We applied a set of predefined criteria to determine which studies qualified for inclusion. We assessed overall survival (OS), progression-free survival (PFS), and cancer-specific survival (CSS) using hazard ratios (HR) and corresponding 95% confidence intervals (CI).

**Results:**

Our analysis included nineteen studies (26 comparative groups), involving 4,422 individuals. Aggregate data revealed a significant correlation between PLR values and both OS and PFS in CRC patients receiving chemotherapy (OS: HR = 1.18, 95% CI: 1.03–1.35; p = 0.02; PFS: HR = 1.28, 95% CI: 1.03–1.60; p = 0.03).

Specifically, higher PLR values were associated with shorter OS and PFS. This association was observed across varying sample sizes, population characteristics, cut-off values, regions, treatments, and patient ages. However, no significant correlation was found between PLR values and CSS in CRC patients receiving chemotherapy (CSS: HR = 1.27, 95% CI: 0.76–2.10; p = 0.36).

**Conclusion:**

Higher PLR values are significantly associated with shorter OS and PFS in CRC patients undergoing chemotherapy. However, the analysis did not demonstrate a statistically significant relationship between PLR and CSS in this patient population. In patients with CRC, PLR may serve as a useful marker for predicting outcomes and shaping individualized therapeutic approaches, especially in the context of immunotherapy.

**Systematic review registration:**

https://www.crd.york.ac.uk/prospero/, identifier CRD420251031290.

## 1 Introduction

Colorectal cancer (CRC) is the third most diagnosed cancer worldwide and the second most common cause of cancer-related deaths, with a noticeable shift toward younger populations (1, 2). Significant advancements have been achieved in both the diagnosis and treatment of CRC. Currently, therapeutic strategies for both early and advanced colorectal cancer are based on a comprehensive approach that includes surgery, radiotherapy, chemotherapy, interventional methods, biotherapy, and photothermal therapy (3). Chemotherapy can be used as an adjuvant or neoadjuvant therapy, aiming to reduce recurrence and improve survival rates (4, 5). This is especially true for patients with stage III CRC (6). The long-term prognosis for CRC patients undergoing chemotherapy varies, influenced by factors such as tumor stage, molecular markers, and the patient’s immune status.

The platelet-to-lymphocyte ratio (PLR) is a novel inflammatory marker calculated by dividing the platelet count by the lymphocyte count (7). In recent years, numerous studies have indicated an association between PLR and the prognosis of CRC patients (6). For instance, An, S. H. et al. (8) examined the association between PLR and 5-year overall survival (OS), disease-free survival (DFS), and the pathological complete response (pCR) rate. Multivariate analysis identified pretreatment PLR as an independent predictor of overall survival (hazard ratio: 1.850; 95% CI: 1.041–3.287; *p* = 0.036). These findings suggest that pretreatment PLR may serve as a prognostic marker for OS in patients with locally advanced rectal cancer undergoing neoadjuvant chemoradiotherapy followed by curative surgery. Sato et al. (9) showed that, in patients with pathological stage I to III CRC, preoperative PLR and pre-stenting PLR were associated with poorer RFS. PLR may reflect the inflammatory and immune status of CRC patients, and elevated PLR often indicates a pro-inflammatory state and immune dysfunction, which are unfavorable for tumor prognosis. Therefore, there is no definite conclusion on the exact prognostic value of PLR for CRC patients receiving chemotherapy.

Several meta-analyses have explored the association between PLR and CRC prognosis. A meta-analysis by Guo et al. (1) incorporated 27 studies encompassing a total of 13,330 patients. The results indicated that elevated PLR levels were significantly associated with poorer OS (HR = 1.40; 95% CI: 1.21–1.62; *p* < 0.00001), DFS (HR = 1.44; 95% CI: 1.09–1.90; *p* = 0.01), and relapse-free survival (RFS) (HR = 1.48; 95% CI: 1.13–1.94; *p* = 0.005). In contrast, no statistically significant associations were observed for progression-free survival (PFS) (HR = 1.14; 95% CI: 0.84–1.54; *p* = 0.40) or cancer-specific survival (CSS) (HR = 1.16; 95% CI: 0.88–1.53; *p* = 0.28). However, Guo et al. did not specifically focus on the association between PLR and prognosis in individuals diagnosed with CRC receiving chemotherapy. Furthermore, after their research was published, new studies yielded conclusions that are not entirely consistent (8, 10, 11). Therefore, the purpose of this meta-analysis was to synthesize this new evidence and specifically investigate the prognostic value of PLR in the distinct and clinically relevant population of CRC patients undergoing chemotherapy.

## 2 Materials and methods

### 2.1 Literature search

Reporting adhered to PRISMA 2025 recommendations (12), with the protocol registered in an international registry for systematic reviews (PROSPERO ID: CRD420251031290). Two researchers, LYF and ZJ, independently formulated the search protocol by selecting relevant subject headings and keywords. The search was performed across several major databases-PubMed, Embase, Web of Science, and the Cochrane Library-covering all available records up to February 20, 2025. A comprehensive set of search terms was employed, including: “Blood Platelet,” “Platelet Blood,” “Platelets Blood,” “Platelets,” “Platelet,” “Thrombocytes,” “Thrombocyte,” “Lymphocyte,” “Lymphoid Cells,” “Cells Lymphoid,” “Cells Lymphoid,” “Cell Lymphoid,” “Lymphoid Cell,” “Colorectal Neoplasm,” “Neoplasm Colorectal,” “Colorectal Tumors,” “Colorectal Tumor,” “Tumor, Colorectal,” “Tumors Colorectal,” “Neplasms Colorectal,” “Colorectal Cancer,” “Cancer, Colorectal “ “Cancers Colorectal,” “Colorectal Cancers,” “Colorectal Carcinoma,” “Carcinoma, Colorectal,” “Carcinomas Colorectal,” “Colorectal Carcinomas,” “Colorectal Neoplasms,” “Therapy Drug,” “Drug Therapies,” “Therapies, Drug,” “Drug Therapy,” “Pharmacotherapies,” “Pharmacotherapy,” “Chemotherapies,” “Chemotherapy,” “ratio,”. The search strategy is comprehensively described in **Supplementary Table S1**.

### 2.2 Study selection

Publications were excluded if they contained duplicate or overlapping data. To be included in this meta-analysis, studies had to meet the following criteria: (1) CRC diagnosis confirmed by pathological examination; (2) Patients received chemotherapy or a combination of chemotherapy and surgery; (3) The study assessed the prognostic relevance of PLR in relation to OS, PFS, or CSS; (4) Sufficient data were provided to obtain a hazard ratio (HR) and 95% confidence interval (CI), either reported directly or calculable from the text; (5) Participants were stratified into high- and low-PLR groups based on predefined cut-off values; (6) A full version of the research was published.

Exclusion criteria included: (1) Reviews, commentaries, conference abstracts, case reports, and letters; (2) Studies that did not provide adequate data to estimate HR and 95% CI; (3) Absence of survival outcome data; (4) Duplicated or overlapping datasets.

Two researchers (LYF and ZJ) independently reviewed the titles and abstracts of studies retrieved from the databases, downloaded full-text articles, and evaluated them to obtain eligible studies. Any disagreements during the study selection process were resolved through consensus.

### 2.3 Data extraction

The extraction of data was carried out by two researchers, LYF and ZJ, independently. Any differences in judgment were addressed and settled collectively by all contributing authors. The obtained details included: the name of the first author, publication year, study period, region, study design, population, treatment method, timing of detection, number of patients, gender, mean age, TNM stage, PLR cut-off and HRs (95% CIs) for OS, PFS, and CSS.

### 2.4 Quality assessment

The quality of the studies was appraised using the Newcastle-Ottawa Scale (NOS), which assesses research based on three criteria: selection, comparability, and outcome, with a maximum achievable score of nine points (13). Studies that received scores ranging from 7 to 9 were classified as high quality (14).

### 2.5 Statistical analysis

The combined HRs and corresponding 95% CIs were calculated to evaluate the prognostic significance of PLR in individuals diagnosed with colorectal cancer and undergoing chemotherapy. Cochran’s Q test and Higgins I (2) statistic were utilized for measuring heterogeneity (15). When I² > 50% or p < 0.1, the heterogeneity was significant.

A random-effects model was employed for all data analysis. Subgroup and sensitivity analyses were performed to confirm the stability and reliability of the findings for OS, PFS, and CSS. To assess potential publication bias, funnel plots were generated, and Egger’s tests were performed, with p-values < 0.05 indicating statistical significance. Statistical analysis was conducted using Review Manager 5.4 and STATA 15.0.

## 3 Results

### 3.1 Study characteristics

**Figure 1:**
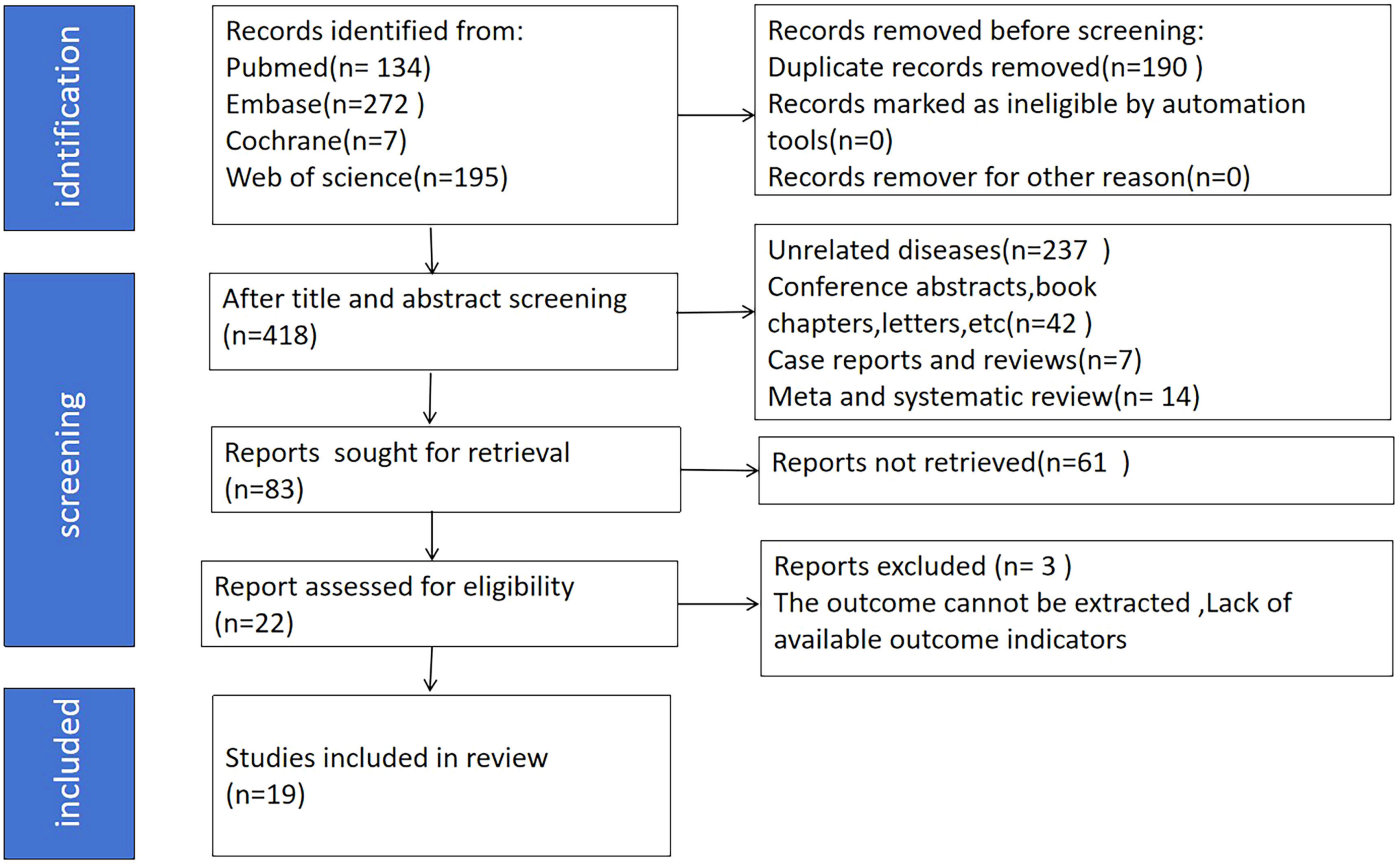
Literature searching and screening process.

The initial database search yielded 608 articles. After removing 190 duplicates, we screened the titles and abstracts of the remaining studies and excluded 300 irrelevant records. Next, we evaluated the full texts of 118 studies. Of these, 64 were excluded mainly because they lacked sufficient data for survival analysis. Ultimately, nineteen studies were included in this meta-analysis (26 comparative groups), encompassing a total of 4,422 patients (3, 5, 6, 8, 10, 11, 16–27). Among the eligible nineteen studies, one was conducted in each of the following countries: Canada, Japan, Spain, USA, Singapore, and Korea. Three studies were conducted in Turkey, while the other ten originated from China. Notably, all included studies were retrospective cohort designs, published in English between 2014 and 2023. Twenty-four comparative groups examined the prognostic implications of PLR on OS, while sixteen comparative groups assessed its prognostic significance for PFS. Six comparative groups analyzed its prognostic significance on CSS. **Table 1** presents the included comparative groups’ characteristics. The literature searching and selection process is depicted in **Figure 1**.

**Table 1:** Basic characteristics of the included literature.

Author	Study period	Region	Study design	Population	Treatment method	Timing of detection	NO. of patients	Gender		Mean/median Age	TNM stage	PLR cut-off	Quality
								Male	Female				
Azab Basem 2014a (16)	2005 - 2011	USA	Retrospective cohort	colorectal cancer	surgery and chemotherapy	before their initial treatment modality	192	NA	NA	69	I-IV	198	7
Azab Basem 2014b (16)							195	NA	NA	NA	NA	244	7
Wu Yuchen 2016 (17)	2008-2013	China	Retrospective cohort	colorectal liver metastasis	surgery and chemotherapy	every 3 months	55	35	20	59	II-IV	150	7
Bong Tiffany Sin Hui 2017 (18)	2003-2015	Singapore	Retrospective cohort	colorectal peritoneal carcinomatosis	cytoreductive surgery and hyperthermic intraperitoneal chemotherapy	1 week before surgery	60	22	38	56	NA	150	7
Zhao Jian 2017 (19)	2006-2013	China	Retrospective cohort	mucinous rectal cancer	chemotherapy	before chemotherapy	100	70	30	60.5	II-III	150	7
Tao Yong 2018 (20)	2009-2012	China	Retrospective cohort	colorectal cancer	surgery and chemotherapy	before any chemotherapy	153	81	72	62	II-IV	186	8
Jing Yang 2018 (21)	2010-2015	China	Retrospective cohort	colorectal cancer	chemotherapy	4 weeks prior to receiving neoadjuvant CRT	98	59	39	53	I-IV	114.5	8
Dogan Ender 2019a (22)	2010-2018	Turkey	Retrospective cohort	metastatic colorectal cancer	chemotherapy(receiving bevacizumab)	before chemotherapy	130	66	64	61	NA	160.66	8
Dogan Ender 2019b (22)	2010-2018	Turkey	Retrospective cohort	metastatic colorectal cancer	chemotherapy(receiving antiEGFR)	before chemotherapy	94	48	46	61	NA	160.66	8
Dudani Shaan 2019	2005-2013	Canadian	Retrospective cohort	locally advanced rectal cancer	surgery and chemotherapy	before chemotherapy	1237	858	379	62	II.III	150	7
Yang Jing 2019 (23)	2009-2015	China	Retrospective cohort	primary stage III/IV CRC	chemotherapy	2 weeks prior to radiotherapy	220	87	133	63	III/IV	151.02	8
Ke Te-Min 2020 (24)	2006-2016	China	Retrospective cohort	rectal cancer	neoadjuvant concurrent chemoradiotherapy CCRT followed by TME	within 2 weeks before neoadjuvant CCRT	184	121	63	64	I.II.III	188	7
Matsuda Akhisa 2020 (25)	2018-2019	Japan	Retrospective cohort	metastatic colorectal cancer	chemotherapy	before chemotherapy	21	15	6	65	II-IV	193.2	7
Zhang Yiyi 2020 (26)	2011-2015	China	Retrospective cohort	locally advanced rectal cancer	surgery and chemotherapy	one week before NCRT	472	313	159	66	NA	169.5	7
Emrah Eraslan 2021 (27)	2014-2020	Turkey	Retrospective cohort	locally advanced rectal cancer	surgery and chemotherapy	before chemotherapy	188	110	78	67	II. III	160	8
Yu Fu 2021a (6)	2010-2015	China	Retrospective cohort	stage II CRC	surgery and chemotherapy	within 1 week before the operation	237	NA	NA	68	NA	130	7
Yu Fu 2021b (6)	2010-2015	China	Retrospective cohort	stage II CRC	surgery and chemotherapy	within 1 week before the operation	210	NA	NA	69	NA	130	7
Yu Fu 2021c (6)	2010-2015	China	Retrospective cohort	colon cancer	surgery and chemotherapy	within 1 week before the operation	NA	NA	NA	70	NA	130	7
Yu Fu 2021d (6)	2010-2015	China	Retrospective cohort	colon cancer	surgery and chemotherapy	within 1 week before the operation	NA	NA	NA	71	NA	130	7
Yu Fu 2021e (6)	2010-2015	China	Retrospective cohort	rectal cancer	surgery and chemotherapy	within 1 week before the operation	NA	NA	NA	72	NA	130	7
Yu Fu 2021f (6)	2010-2015	China	Retrospective cohort	rectal cancer	surgery and chemotherapy	within 1 week before the operation	NA	NA	NA	73	NA	130	7
Jia Wangqiang 2021 (3)	2011-2014	China	Retrospective cohort	colorectal cancer	surgery and chemotherapy	before the operation	145	89	56	74	II. III	154.31	7
P. Wang 2021 (28)	2013-2016	China	Retrospective cohort	Rectal cancer	surgery and chemotherapy	before the operation	75	46	29	75	III/IV	7.02	7
An Sang Hyun 2022 (8)	1996-2015	Korea	Retrospective cohort	locally ad vanced rectal cancer	surgery and chemotherapy	1 week prior to neoadjuvant CRTand index surgery.	162	121	41	76	0-III	170	7
Bulut Gulcan 2022 (10)	2010-2020	Turkey	Retrospective cohort	metastatic colorectal cancer	surgery and chemotherapy	1 week before the first-line chemotherapy.	94	56	38	77	NA	180.36	9
Duque-SantanaVictor 2023 (11)	2012-2017	Spain	Retrospective cohort	locally advanced rectal cancer	surgery and chemotherapy	1–10 days prior to nCRT	100	57	43	78	0-III	133	7

CRC, Colorectal cancer; NOS, Newcastle-Ottawa scale; OS, Overall survival; PFS, Progression-free survival; PLR, platelet to lymphocyte ratio; NA, not available.

### 3.2 Study quality

All studies received Newcastle-Ottawa Scale (NOS) scores ranging from 7 to 9, reflecting high methodological quality (**Supplementary Table S2**).

### 3.3 Meta-analysis results

#### 3.3.1 PLR and OS

**Figure 2:**
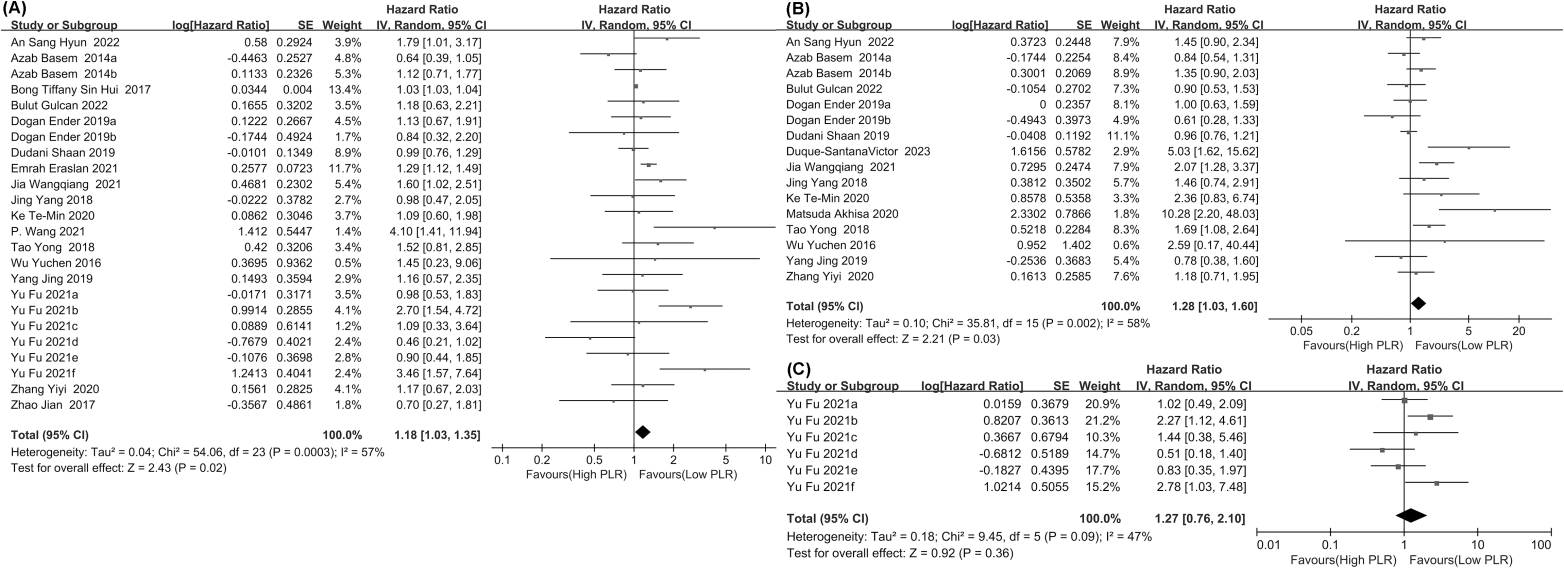
Forest plots **(A)** for the association between PLR and OS, **(B)** for the association between PLR and PFS, **(C)** for the association between PLR and CSS.

We examined the association between PLR and OS across 24 comparison groups from cohort studies including 4,301 patients. The analysis demonstrated a strong association between higher PLR levels and poorer OS in CRC patients undergoing chemotherapy (HR = 1.18, 95% CI: 1.03–1.35; *p* = 0.02, **Figure 2A**), indicating that a higher PLR is associated with reduced survival in this population.

To explore potential sources of heterogeneity, subgroup analyses were performed based on population characteristics, PLR cut-off values, geographic region, treatment methods, median age, and sample size. The results of these analyses are summarized in **Table 2**. These factors were all sources contributing to OS heterogeneity. It was found that in cases of metastatic colorectal cancer, when the PLR cut-off was < 150, in studies conducted in America, with chemotherapy as the treatment method, and where the median age was ≥60 years or <60 years, and the number of patients was ≥150 or <150, PLR had no significant predictive value for OS, but it was significant in other subgroups.

**Table 2:** Pooled HRs for OS and PFS in subgroup analyses.

Subgroup	OS(Baseline)				PFS(Baseline)			
	Study group	HR [95%CI]	P value	I2	Study group	HR [95%CI]	P value	I2
Total	24	1.18[1.03-1.35]	0.02	57%	16	1.28 [1.03-1.60]	0.03	58%
Population								
metastatic colorectal cancer	4	1.11 [0.77-1.60]	0.57	0%	5	1.17 [0.63-2.17]	0.62	63%
colorectal cancer	20	1.19 [1.03-1.39]	0.02	64%	11	1.34 [1.06-1.70]	0.01	57%
PLR cut-off								
≥150	16	1.12 [1.01-1.26]	0.04	36%	14	1.21 [0.98-1.50]	0.08	55%
<150	8	1.40 [0.83-2,36]	0.2	73%	2	2.49 [0.75-8.25]	0.14	70%
Region								
Asia	19	1.40 [1.03-1.04]	<0.00001	60%	12	1.33 [1.01-1.73]	0.01	51%
America	3	0.94 [0.76-1.16]	0.56	34%	3	1.02 [0.80-1.29]	0.89	29%
Europe	NS	NS	NS	NS	1	5.03 [1.62-15.62]	0.005	NS
treatment method								
chemotherapy	5	1.01 [0.73-1.4]	0.94	0%	5	1.17 [0.66-2.07]	0.59	66%
surgery and chemotherapy	19	1.04 [1.03-1.04]	<0.00001	66%	11	1.34 [1.05-1.70]	0.02	57%
Mean/median age								
≥60y	17	1.16 [0.96-1.40]	0.12	55%	11	1.26 [0.96-1.65]	0.09	65%
<60y	7	1.14 [0.96-1.40]	0.1	42%	5	1.37 [0.95-1.97]	0.09	31%
Number of patients								
≥150	15	1.19 [0.98-1.46]	0.08	58%	8	1.21 [0.98-1.49]	0.07	24%
<150	9	1.15 [0.94-1.42]	0.18	29%	8	1.51 [1.95-2.40]	0.08	74%

#### 3.3.2 PLR and PFS

We examined the association between PLR and PFS across sixteen comparative groups from cohort studies including 3,552 patients. The analysis revealed a significant link between higher PLR levels and poorer PFS in CRC patients undergoing chemotherapy (HR = 1.28, 95% CI: 1.03–1.60; *p* = 0.03, **Figure 2B**), indicating that higher PLR is linked to a shorter progression-free survival.

To explore sources of heterogeneity, subgroup analyses were performed based on population, PLR cut-off values, region, treatment modality, median age, and sample size. The results of these analyses are presented in **Table 2**. The main sources of heterogeneity included treatment method, median age, and number of patients. It was found that in metastatic colorectal cancer, when the PLR cut-off was <150 or ≥150, in studies from America, with chemotherapy as the treatment method, and where the median age was ≥60 years or <60 years, and the number of patients was ≥150 or <150, PLR had no significant predictive value for PFS, but it was significant in other subgroups.

#### 3.3.3 PLR and CSS

We investigated the relationship between PLR and CSS, involving six comparative groups that explored its prognostic significance in cohort studies comprising 447 participants. The analysis showed no significant association between PLR levels and CSS in CRC patients undergoing chemotherapy (HR = 1.27, 95% CI: 0.76–2.10; p = 0.36, **Figure 2C**).

### 3.4 Sensitivity analysis

**Figure 3:**
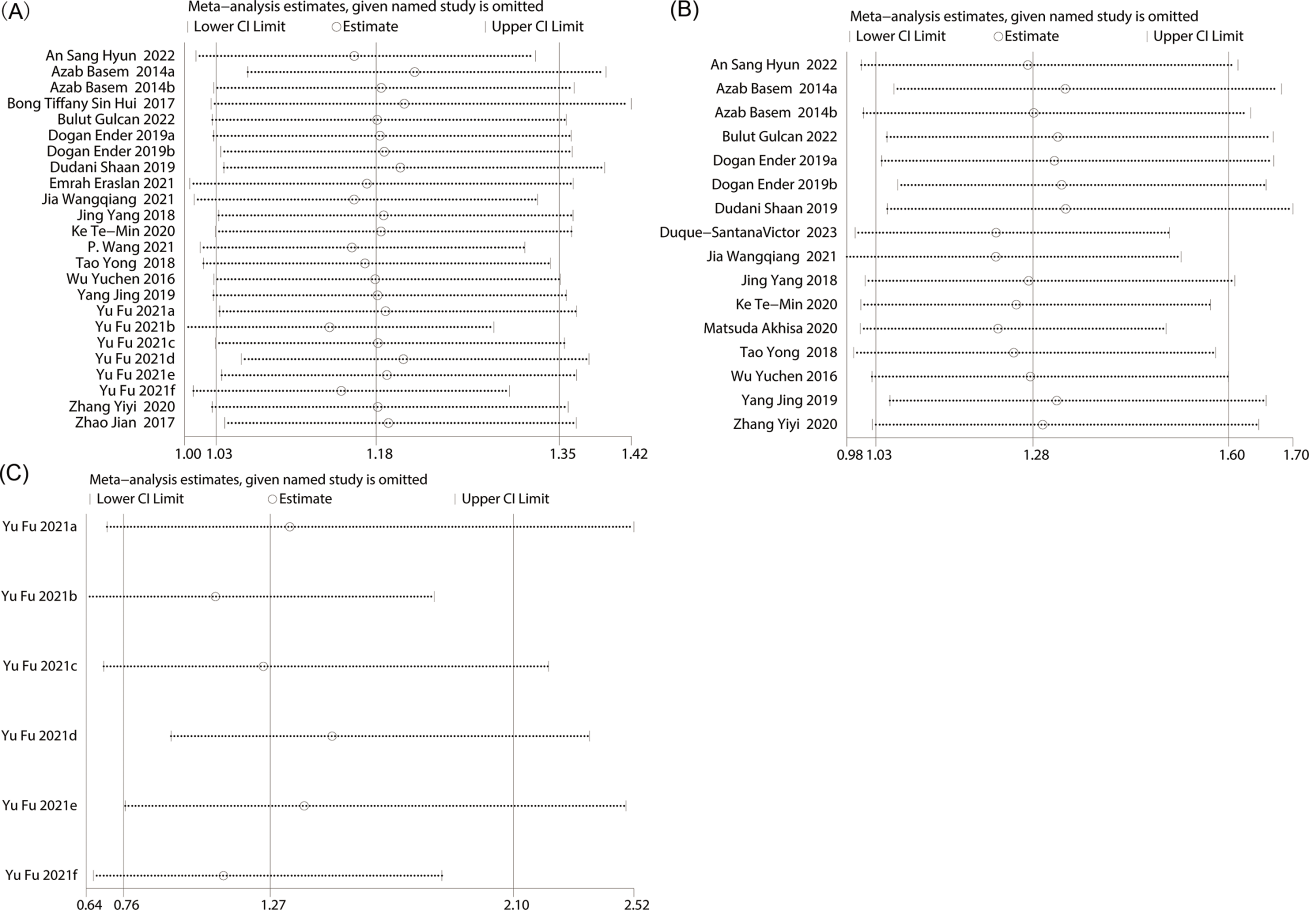
Sensitivity analysis of **(A)** OS, **(B)** PFS and **(C)** CSS.

A sensitivity analysis was performed to assess the stability of the findings regarding the prognostic value of baseline PLR. The results demonstrated that the effect size remained stable within the original range after sequential exclusion of each study, indicating that no individual study had an undue impact on the overall outcomes for OS (**Figure 3A**), thus confirming the robustness of the results. In the sensitivity analysis of PFS, it was shown that when the three articles (3, 11, 20) were excluded, the predictive value of PLR for PFS changed from significant to non-significant. This indicates that there is instability in the prediction of PFS indicators by PLR (**Figure 3B**). In the sensitivity analysis of CSS, it was shown that there is stability in the prediction of CSS indicators by PLR (**Figure 3C**).

### 3.5 Publication bias

**Figure 4:**
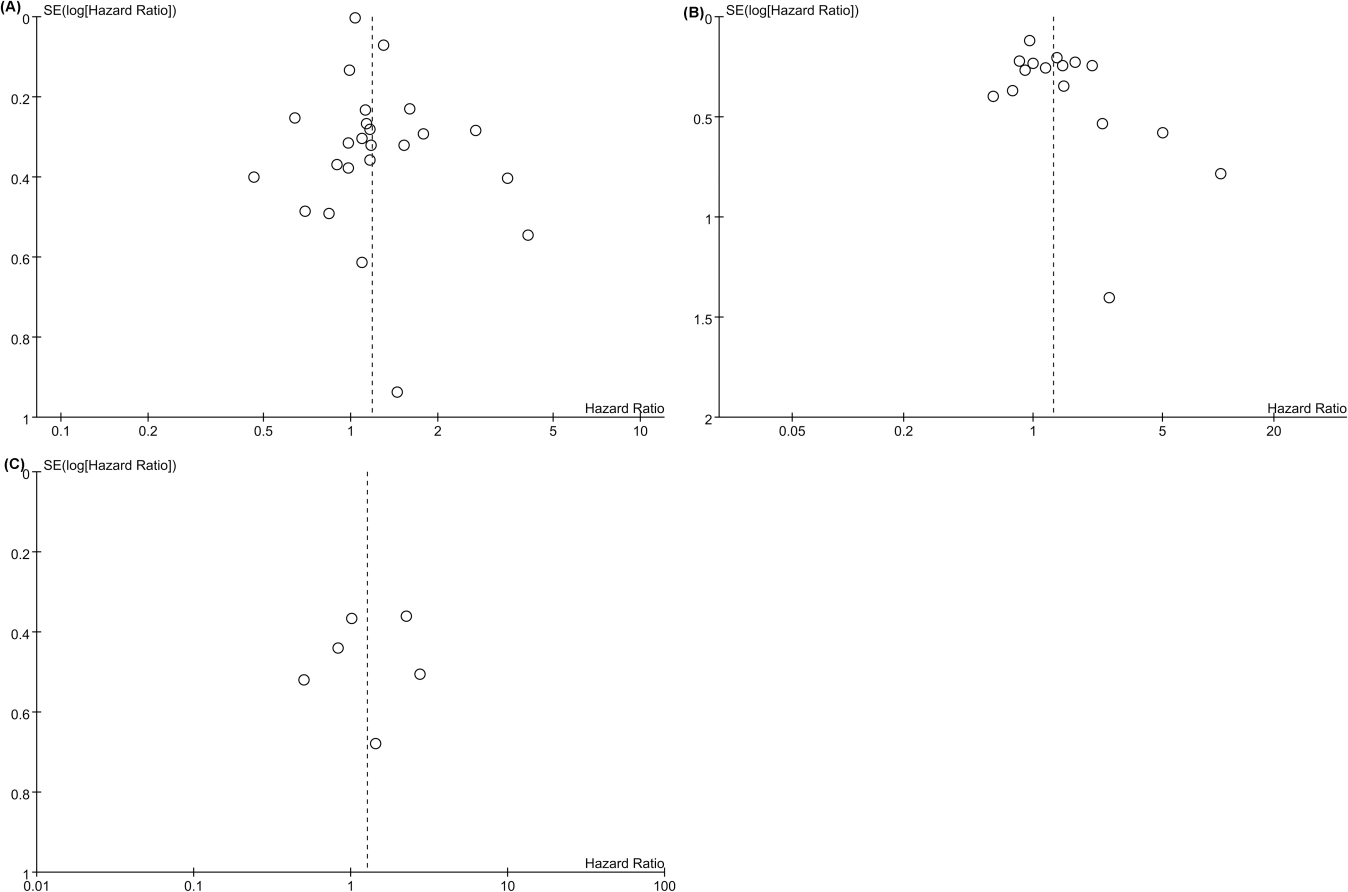
Funnel plot for the evluation of publication bias for **(A)** OS, **(B)** PFS and **(C)** CSS.

Publication bias was assessed through Egger’s test and visual inspection of funnel plots. The Egger’s test results showed no significant publication bias for OS (*p* = 0.075), PFS (*p* = 0.051), or CSS (*p* = 0.775). Additionally, the symmetrical appearance of the funnel plot further supported the lack of substantial publication bias in the meta-analysis for OS (**Figure 4A**), PFS (**Figure 4B**), CSS (**Figure 4C**).

## 4 Discussion

Inflammation plays a pivotal role as one of the primary contributors to colorectal carcinogenesis (28). Among various biomarkers, the PLR has shown potential in predicting survival outcomes in patients with CRC (29). Research by Hong-Xin Peng found that higher PLR levels correlate with poor OS, particularly in metastatic CRC patients of Caucasian descent. A meta-analysis including 12 studies with 3,541 patients further supported this association, revealing that a high PLR significantly affects OS, DFS, and CSS in both the overall population and the Caucasian subgroup. Interestingly, increased PLR was not correlated with DFS in individuals treated solely with surgery, but it was strongly linked to poorer survival outcomes in metastatic CRC patients. This suggests that PLR has prognostic relevance for OS, DFS, and PFS in the metastatic subgroup. While these findings underscore the potential of PLR as a prognostic biomarker, especially in metastatic Caucasian patients, further research is needed to validate these associations and confirm its clinical utility (30).

In recent years, inflammatory indicators such as PLR, NLR (31, 32), and LMR are of great significance in the diagnosis and prognosis of various cancers, including CRC (11, 33). The relationship between PLR and CRC prognosis, confirmed by a growing body of research, has therefore drawn considerable attention. In our study, we conducted a meta-analysis including 19 studies (26 comparative groups), encompassing a total of 4,422 patients, to assess the prognostic value of PLR in CRC survival. Unlike previous meta-analyses that examined PLR across all treatment modalities, our study specifically focused on patients receiving chemotherapy, allowing for a more tailored assessment of its prognostic utility in this subpopulation. Our results demonstrated a significant association between higher PLR and both OS and PFS. However, no statistically significant association was found between PLR and CSS. One possible explanation for the lack of significance regarding CSS is that these data were derived from different subgroups within the same studies. Additional high-quality research is warranted to further evaluate whether PLR can reliably predict CSS in CRC patients.

Sensitivity analysis revealed that PLR for predicting PFS in CRC patients undergoing chemotherapy was unstable. This suggests the need for further research to confirm the relationship between PLR and PFS. Nonetheless, evaluation of publication bias using Egger’s test and funnel plots indicated no significant risk of bias for the three outcomes-OS, PFS, and CSS-suggesting a relatively high level of evidence. Our meta-analysis demonstrated that higher PLR levels are significantly associated with worse OS (1). Interestingly, even though Ganlin Guo’s study did not stratify patients based on treatment modalities, PLR remained predictive of OS, indicating that the type of treatment may not significantly influence the prognostic value of PLR. Despite this, the association between PLR and PFS prediction is inconclusive. While Ganlin Guo concluded that PLR had no significant prognostic value for either PFS or CSS, our study found a significant correlation between elevated PLR and PFS, though not with CSS. A key difference between the two studies lies in the inclusion criteria: our study limited the analysis to patients undergoing chemotherapy, whereas Guo’s analysis did not. This specific focus on a treatment-defined population likely explains the divergent finding regarding PFS, suggesting that chemotherapy may modulate the relationship between PLR and disease progression. Furthermore, our meta-analysis incorporated several additional methodological approaches—such as subgroup analysis, sensitivity analysis, and publication bias assessment via funnel plots—to evaluate evidence quality more rigorously. These methods allowed us to explore the optimal application conditions and target populations for PLR, thereby improving the robustness of the research.

In our systematic review and meta-analysis, subgroup analyses revealed that PLR lacked prognostic significance in specific contexts: metastatic colorectal cancer, PLR cut-off values <150, populations from the United States, and patients treated with chemotherapy alone. Several epidemiological and clinical explanations may account for these observations. Firstly, in metastatic CRC, the disease has reached an advanced phase characterized by extensive pathological changes, a high tumor burden, and a compromised immune system. At this point, systemic inflammation and immune responses may be highly dysregulated or have reached a plateau. Consequently, PLR, which reflects the balance between platelets and lymphocytes, may no longer serve as a reliable indicator of prognosis. Some studies have shown that in advanced cancers, complex immunosuppressive mechanisms can obscure the predictive value of simple inflammatory markers such as PLR. Secondly, regarding the PLR cut-off value <150, the prognostic utility of PLR depends heavily on the threshold used to define high and low levels. Different studies adopt various methods to determine cut-off values-e.g., medians, receiver operating characteristic (ROC) curve analysis, or other statistical criteria. When a relatively low threshold such as <150 is used, it may not sufficiently differentiate high-risk patients, particularly in cohorts with generally lower PLR distributions. In such cases, the prognostic difference between high and low PLR groups may be negligible, leading to an apparent lack of significance. Indeed, some studies suggest that using higher cut-off values may improve the predictive performance of PLR in survival analysis.

Thirdly, regional differences-particularly in American populations-may also contribute to the observed variation in the prognostic value of PLR. Genetic, environmental, and lifestyle factors vary significantly across regions and can influence both the epidemiology of colorectal cancer and its associated biomarkers. In the American population, a high degree of genetic heterogeneity exists, alongside unique environmental exposures, dietary patterns, and disparities in healthcare access. These factors can collectively affect the host’s immune-inflammatory response, potentially altering the relationship between PLR and patient prognosis. Moreover, differences in clinical practice and treatment protocols across countries may further impact the prognostic utility of PLR. For example, American treatment guidelines for colorectal cancer-including chemotherapy regimens, the use of targeted therapies, and follow-up strategies-may differ from those used in Asian or European countries. These regional disparities may reduce the consistency of PLR’s prognostic value when applied across diverse populations.

“Fourth, the geographical distribution of the included studies is a significant limitation. The majority of studies were conducted in China, which may introduce regional bias and limit the generalizability of our findings to other ethnic and geographical populations, particularly those of African or European ancestry. The biological behavior of CRC, host inflammatory responses, and treatment protocols can vary across regions. Therefore, extrapolation of our results to a global context should be done with caution. Future multicenter studies involving more diverse populations are urgently needed to validate the universal prognostic value of PLR.

Lastly, regarding treatment modality-chemotherapy alone. Patients receiving only chemotherapy may generally be in advanced stages of the disease, where the tumor has already metastasized and lost the opportunity for surgical resection. In such cases, the tumor’s biological behavior and the body’s immune-inflammatory response may differ from those in early-stage patients. Chemotherapy drugs primarily target tumor cells but may also suppress the immune system to some extent. This could further complicate the relationship between PLR and prognosis. In contrast, early-stage colorectal cancer patients with relatively intact immune function may exhibit a clearer association between PLR and prognosis. Some studies have also noted that the combination of chemotherapy and other treatments, such as targeted therapy, may alter the prognostic significance of PLR. In clinical practice, the following recommendations can be made based on these findings: when using PLR to assess the prognosis of colorectal cancer patients, it is advisable to select a PLR cut-off value of ≥150 to enhance its predictive accuracy. For metastatic colorectal cancer patients or those undergoing chemotherapy alone, PLR should be interpreted with caution, as its prognostic value may be limited. Greater emphasis should be placed on combining PLR with other clinical indicators and molecular markers to comprehensively evaluate prognosis. Furthermore, considering regional differences, treatment guidelines and practices for colorectal cancer should be developed and optimized based on local epidemiological characteristics. This will help improve the precision of prognosis prediction and provide more individualized treatment strategies. In conclusion, the prognostic value of PLR in individuals diagnosed with colorectal cancer is influenced by multiple factors, including disease stage, PLR cut-off value, geographic region, and treatment modality. Additional studies are needed to understand the mechanisms underlying these associations and to establish more appropriate PLR cut-off values and application scenarios for different populations. This will enable the effective use of PLR in clinical prognosis prediction.

Studies have shown the significance of the PLR as an indicator of the body’s inflammatory response, which is intricately connected to tumor development (34, 35). Inflammation, a fundamental component of the immune system, can create a favorable environment for cancer progression. Within the tumor microenvironment, a complex interplay of cellular interactions occurs, involving inflammatory cells and platelets that release a variety of substances contributing to cancer development (36, 37). Inflammatory cells secrete bioactive factors, such as cytokines and chemokines, which modulate the immune response and contribute to the creation of a pro-tumorigenic environment. These factors are essential for initiating and sustaining the inflammatory processes that support tumor growth. Platelets, in turn, release several key substances, including adhesion molecules that facilitate cancer cell attachment to the extracellular matrix, angiogenic factors that stimulate the formation of new blood vessels to nourish the growing tumor, and tumor-promoting factors that enhance cancer cell survival and proliferation (38, 39). Collectively, these substances drive tumor formation, angiogenesis, cancer cell invasion, metastasis, and overall tumor progression (40, 41). In the context of colorectal cancer-a common malignancy associated with significant morbidity and mortality-PLR levels have been shown to increase progressively as the disease advances (1). Elevated PLR values correlate with poorer prognosis, indicating that PLR may be a useful prognostic marker. Monitoring PLR levels can provide clinicians with a simple and non-invasive tool to assess disease progression and guide treatment decisions. This biomarker offers a potential means to identify patients at higher risk of poor outcomes, enabling earlier interventions and potentially improving survival rates. Further research into the mechanisms underlying the association between PLR and cancer progression may facilitate the development of new therapeutic strategies targeting inflammation and tumor microenvironment interactions.

Our results provide a more accurate reflection of the association between PLR and CRC prognosis. However, several limitations of our study should be acknowledged. First, much of the data comes from China, and thus the risk of selection bias cannot be eliminated. Second, the sample size was relatively small, which limits the generalizability of our results. We acknowledge that our research is not without flaws; nevertheless, it offers clinically relevant insights into the prognostic value of PLR in colorectal cancer. We look forward to future high-quality, multicenter RCTs to minimize these biases and further validate our findings.

We employed the term ‘colorectal cancer (CRC)’ to reflect the broader patient population encompassed by the included studies, many of which grouped colon and rectal cancers together. We acknowledge the distinct anatomical and clinical characteristics between colon and rectal cancer, which may influence outcomes. The prognostic value of PLR might differ between these two sub-types based on their specific tumor microenvironments and treatment paradigms (e.g., the central role of neoadjuvant chemoradiation in rectal cancer). Unfortunately, the primary studies did not provide sufficient disaggregated data to perform a subgroup analysis based on the primary tumor site. This represents a limitation of our analysis and highlights an important area for future research. Future prospective studies should aim to investigate the prognostic role of PLR in colon cancer and rectal cancer cohorts separately.

## 5 Conclusion

Our meta-analysis revealed that PLR is a significant predictor of OS and PFS in CRC patients undergoing chemotherapy. Specifically, higher PLR values were associated with shorter survival times in this patient group. Further subgroup analyses indicated that several factors-including population characteristics, PLR cut-off values, geographic region, treatment methods, patient age, and sample size-may influence the predictive efficacy of PLR in this context.

However, it should be emphasized that most of the studies included were retrospective in nature, had relatively small sample sizes, and may have limitations regarding result stability. Therefore, we recommend that future research focus on prospective, multicenter studies to further validate the prognostic value of PLR in CRC patients receiving chemotherapy. Such studies will help provide more robust and reliable evidence to support the clinical application of PLR as a prognostic biomarker in this patient population.

## Data Availability

The original contributions presented in the study are included in the article/**Supplementary Material**. Further inquiries can be directed to the corresponding author.
