# Supplementary material for: Prognostic value of platelet to lymphocyte ratio in patients with colorectal cancer undergoing chemotherapy: a systematic review and meta-analysis
Source: Front Immunol. 2025 Nov 3;16:1642181. doi: 10.3389/fimmu.2025.1642181 (PMC12620380; doi:10.3389/fimmu.2025.1642181)
Supplement: Supplementary file 2 [file Table2.docx]

Supplementary Material

| Table S1 Detailed search strategy in four databases | |
| --- | --- |
| Database | Search strategy |
| Pubmed | (ratio) AND (((((((((((Chemotherapy) OR (Chemotherapies)) OR (Phrmacotherapy)) OR (Pharmacotherapies)) OR (Therapy, Drug)) OR (Drug Therapies)) OR (Therapies, Drug)) OR ("Drug Therapy"[Mesh])) AND ((((((((((((((((Colorectal Neoplasm) OR (Neoplasm, Colorectal)) OR (Colorectal Tumors)) OR (Colorectal Tumor)) OR (Tumor, Colorectal)) OR (Tumors, Colorectal)) OR (Neplasms, Colorectal)) OR (Colorectal Cancer)) OR (Cancer, Colorectal)) OR (Cancers, Colorectal)) OR (Colorectal Cancers)) OR (Colorectal Carcinoma)) OR (Carcinoma, Colorectal)) OR (Carcinomas, Colorectal)) OR (Colorectal Carcinomas)) OR ("Colorectal Neoplasms"[Mesh]))) AND ((((((Lymphocyte) OR (Lymphoid Cells)) OR (Cell, Lymphoid)) OR (Cells, Lymphoid)) OR (Lymphoid Cell)) OR ("Lymphocytes"[Mesh]))) AND (((((((Blood Platelet) OR (Platelet, Blood)) OR (Platelets)) OR (Platelet)) OR (Thrombocytes)) OR (Thrombocyte)) OR ("Blood Platelets"[Mesh]))) |
| Embase | (ratio) AND (((((((((((Chemotherapy) OR (Chemotherapies)) OR (Phrmacotherapy)) OR (Pharmacotherapies)) OR (Therapy Drug)) OR (Drug Therapies)) OR (Therapies Drug)) OR (Drug Therapy)) AND ((((((((((((((((Colorectal Neoplasm) OR (Neoplasm Colorectal)) OR (Colorectal Tumors)) OR (Colorectal Tumor)) OR (Tumor Colorectal)) OR (Tumors Colorectal)) OR (Neoplasms Colorectal)) OR (Colorectal Cancer)) OR (Cancer, Colorectal)) OR (Cancers Colorectal)) OR (Colorectal Cancers)) OR (Colorectal Carcinoma)) OR (Carcinoma Colorectal)) OR (Carcinomas Colorectal)) OR (Colorectal Carcinomas)) OR (Colorectal Neoplasms))) AND ((((((Lymphocyte) OR (Lymphoid Cells)) OR (Cell, Lymphoid)) OR (Cells, Lymphoid)) OR (Lymphoid Cell)) OR (Lymphocytes))) AND (((((((Blood Platelet) OR (Platelet Blood)) OR (Platelets)) OR (Platelet)) OR (Thrombocytes)) OR (Thrombocyte)) OR (Blood Platelets))) |
| Web of Science | (ratio) AND (((((((((((Chemotherapy) OR (Chemotherapies)) OR (Phrmacotherapy)) OR (Pharmacotherapies)) OR (Therapy Drug)) OR (Drug Therapies)) OR (Therapies Drug)) OR (Drug Therapy)) AND ((((((((((((((((Colorectal Neoplasm) OR (Neoplasm Colorectal)) OR (Colorectal Tumors)) OR (Colorectal Tumor)) OR (Tumor Colorectal)) OR (Tumors Colorectal)) OR (Neoplasms Colorectal)) OR (Colorectal Cancer)) OR (Cancer, Colorectal)) OR (Cancers Colorectal)) OR (Colorectal Cancers)) OR (Colorectal Carcinoma)) OR (Carcinoma Colorectal)) OR (Carcinomas Colorectal)) OR (Colorectal Carcinomas)) OR (Colorectal Neoplasms))) AND ((((((Lymphocyte) OR (Lymphoid Cells)) OR (Cell, Lymphoid)) OR (Cells, Lymphoid)) OR (Lymphoid Cell)) OR (Lymphocytes))) AND (((((((Blood Platelet) OR (Platelet Blood)) OR (Platelets)) OR (Platelet)) OR (Thrombocytes)) OR (Thrombocyte)) OR (Blood Platelets))) |
| Chochrane | (ratio) AND (((((((((((Chemotherapy) OR (Chemotherapies)) OR (Phrmacotherapy)) OR (Pharmacotherapies)) OR (Therapy Drug)) OR (Drug Therapies)) OR (Therapies Drug)) OR (Drug Therapy)) AND ((((((((((((((((Colorectal Neoplasm) OR (Neoplasm Colorectal)) OR (Colorectal Tumors)) OR (Colorectal Tumor)) OR (Tumor Colorectal)) OR (Tumors Colorectal)) OR (Neoplasms Colorectal)) OR (Colorectal Cancer)) OR (Cancer, Colorectal)) OR (Cancers Colorectal)) OR (Colorectal Cancers)) OR (Colorectal Carcinoma)) OR (Carcinoma Colorectal)) OR (Carcinomas Colorectal)) OR (Colorectal Carcinomas)) OR (Colorectal Neoplasms))) AND ((((((Lymphocyte) OR (Lymphoid Cells)) OR (Cell, Lymphoid)) OR (Cells, Lymphoid)) OR (Lymphoid Cell)) OR (Lymphocytes))) AND (((((((Blood Platelet) OR (Platelet Blood)) OR (Platelets)) OR (Platelet)) OR (Thrombocytes)) OR (Thrombocyte)) OR (Blood Platelets))) |

| Table S2 Quality evaluation of the eligible studies with Newcastle–Ottawa scale | | | | | | | | | |
| --- | --- | --- | --- | --- | --- | --- | --- | --- | --- |
| Study | Selection | | | | Comparability | | Outcome | | |
|  | Representative-ness | Selection of  non-exposed | Ascertainment  of exposure | Outcome not present at start | Comparability on most important factors | Comparability on other risk factors | Assessment of outcome | Long enough follow-up (median≥1 year) | Adequacy  (completeness) of follow-up |
| Azab Basem 2014 | * | * | * | * | - | - | * | * | * |
| Wu Yuchen 2016 | * | * | * | * | - | - | * | * | * |
| Bong Tiffany Sin Hui 2017 | * | * | * | * | - | - | * | * | * |
| Zhao Jian 2017 | * | * | * | * | - | - | * | * | * |
| Tao Yong 2018 | * | * | * | * | * | - | * | * | * |
| Yang Jing 2018 | * | * | * | * | * | - | * | * | * |
| Dogan Ender 2019 | * | * | * | * | * | - | * | * | * |
| Dudani Shaan 2019 | * | * | * | * | - | - | * | * | * |
| Yang Jing 2019 | * | * | * | * | - | * | * | * | * |
| Te-Min Ke 2020 | * | * | * | * | - | - | * | * | * |
| Matsuda Akhisa 2020 | * | * | * | * | - | - | * | * | * |
| Zhang Yiyi 2020 | * | * | * | * | - | - | * | * | * |
| Eraslan Emrah 2021 | * | * | * | * | * | - | * | * | * |
| Fu Yu 2021 | * | * | * | * | - | - | * | * | * |
| Jia Wangqiang2021 | * | * | * | * | - | - | * | * | * |
| Wang P 2021 | * | * | * | * | - | - | * | * | * |
| An Sang Hyun 2022 | * | * | * | * | - | - | * | * | * |
| Bulut Gulcan 2022 | * | * | * | * | * | * | * | * | * |
| Duque-Santana Victor 2023 | * | * | * | * | - | - | * | * | * |
| *indicates criterion met; - indicates significant of criterion not met. | | | | | | | | | |
